# Cardio-oncology rehabilitation and exercise: evidence, priorities, and research standards from the ICOS-CORE working group

**DOI:** 10.1093/eurheartj/ehaf100

**Published:** 2025-02-28

**Authors:** Scott C Adams, Fernando Rivera-Theurel, Jessica M Scott, Michelle B Nadler, Stephen Foulkes, Darryl Leong, Tormod Nilsen, Charles Porter, Mark Haykowsky, Husam Abdel-Qadir, Sarah C Hull, Neil M Iyengar, Christina M Dieli-Conwright, Susan F Dent, Erin J Howden

**Affiliations:** Cancer Fatigue Services Inc., Toronto, ON, Canada; Ted Rogers Cardiotoxicity Prevention Program, University Health Network, Toronto, ON, Canada; Faculty of Medicine, University of Toronto, Toronto, ON, Canada; Cardiovascular Prevention and Rehabilitation Program, Toronto Rehabilitation Institute, University Health Network, Toronto, ON, Canada; Department of Medicine, Memorial Sloan Kettering Cancer Center, New York, NY, USA; Division of Medical Oncology and Hematology, Department of Medicine, University of Toronto, Toronto, ON, Canada; Princess Margaret Cancer Center, University Health Network, Toronto, ON, Canada; Faculty of Nursing, College of Health Sciences, University of Alberta, Edmonton, AB, Canada; The Population Health Research Institute and Department of Medicine, McMaster University and Hamilton Health Sciences, Hamilton, ON, Canada; Department of Physical Performance, Norwegian School of Sports Sciences, Oslo, Norway; Department of Cardiovascular Medicine, University of Kansas Medical Center, Kansas City, KS, USA; Faculty of Nursing, College of Health Sciences, University of Alberta, Edmonton, AB, Canada; Ted Rogers Cardiotoxicity Prevention Program, University Health Network, Toronto, ON, Canada; Faculty of Medicine, University of Toronto, Toronto, ON, Canada; Women’s College Hospital and Peter Munk Cardiac Centre, Toronto, ON, Canada; Section of Cardiovascular Medicine, Yale School of Medicine, New Haven, CT, USA; Program for Biomedical Ethics, Yale School of Medicine, New Haven, CT, USA; Breast Medicine Service, Memorial Sloan Kettering Cancer Center, New York, NY, USA; Department of Medicine, Weill Cornell Medicine, New York, NY, USA; Division of Population Sciences, Department of Medical Oncology, Dana-Farber Cancer Institute, Boston, MA, USA; Harvard Medical School, Harvard University, Boston, MA, USA; Department of Nutrition, Harvard T.H. Chan School of Public Health, Harvard University, Boston, MA, USA; Wilmot Cancer Institute, Department of Medicine, University of Rochester, Rochester, NY, USA; Cardiometabolic Health and Exercise Physiology Laboratory, Baker Heart and Diabetes Institute, 75 Commercial Road, Melbourne, VIC 3004, Australia

## Abstract

The aim of this whitepaper is to review the current state of the literature on the effects of cardio-oncology rehabilitation and exercise (CORE) programmes and provide a roadmap for improving the evidence-based to support the implementation of CORE. There is an urgent need to reinforce and extend the evidence informing the cardiovascular care of cancer survivors. CORE is an attractive model that is potentially scalable to improve the cardiovascular health of cancer survivors as it leverages many of the existing frameworks developed through decades of delivery of cardiac rehabilitation. However, there are several challenges within this burgeoning field, including limited evidence of the efficacy of this approach in patients with cancer. In this paper, a multidisciplinary team of international experts highlights priorities for future research in this field and recommends standards for the conduct of research.

## Graphical Abstract

**Graphical Abstract:**
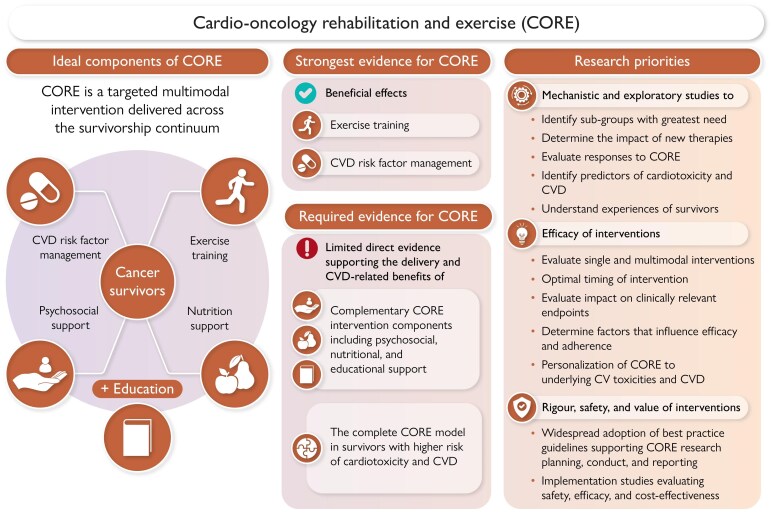
Components, evidence, and research priorities for cardio-oncology rehabilitation and exercise. CV, cardiovascular; CVD, cardiovascular disease.

## Whitepaper background and development

*Background*: Advancements in our understanding of cancer biology, treatments, and supportive care strategies, have improved outcomes leading to more individuals surviving a cancer diagnosis.^1,2^ In high-income countries like the United States (US), the population of cancer survivors is expected to increase from 18.1 million in 2022 to 22.5 million by 2032.^3^ In 2022, 47% of US cancer survivors have lived ≥10 years, and 18% of survivors have lived ≥20 years post-diagnosis.^3^ However, the quality and duration of survival for cancer survivors are often diminished due to the development or worsening of comorbidity, and cancer treatment-related injury, or dysfunction of the cardiopulmonary, skeletal muscle, and metabolic systems.^4,5^ Collectively, these sequelae can manifest in survivors as cardiovascular system damage and dysfunction (known as cardiovascular toxicity), leading to an increased risk of premature mortality and morbidity from cardiovascular disease (CVD).^6,7^ The adverse cardiovascular and metabolic consequences of cancer therapies have been recognized since the mid-1950s^8,9^; yet, the mainstream emergence of cardio-oncology (i.e. an intersectional specialist field aimed at improving cardiovascular care for cancer survivors)^10^ has only emerged in the last two decades. More recently, adverse oncologic outcomes have been associated with prior occurrence of cardiovascular events^11^ and the presence of CVD risk factors,^12^ suggesting that the relationship between cancer and CVD may be bidirectional.^13^ Ultimately, the existence of common mechanisms and risk factors underlying cancer and CVD development and their adverse reciprocal influence on each other^14^ make a strong argument for establishing robust screening and treatment methods that help prevent, mitigate, or reverse these sequelae.

Epidemiological data suggest that modifiable metabolic and behavioural risk factors explain at least 50% of attributable CVD risk.^15^ Understandably, the promotion of a healthy lifestyle (e.g. structured exercise training, recreational physical activity, healthy eating habits, non-smoking) is a cornerstone of CVD prevention^16^ and rehabilitation and, accordingly, has been endorsed in the latest position statements as an integral component of cardio-oncology care.^17–19^ The potential benefits of exercise-based screening to identify at-risk individuals (e.g. via CPET, cardiopulmonary exercise test)^18^ and to intervene with multimodal interventions (e.g. cardiac rehabilitation^19^) have been highlighted as priorities for research and patient care. Cardiac rehabilitation is an exercise-based multimodal care model that incorporates parallel nutrition, CVD risk factor management, education (e.g. medication adherence), behavioural, and psychosocial intervention components,^20^ with demonstrated benefits including improving CVD risk, cardiorespiratory fitness, health-related quality of life (HRQoL), and survival across multiple clinical populations. Recent American Heart Association (AHA) and American Cancer Society (ACS) recommendations^19^ endorsed the adaptation and adoption of this comprehensive care model (i.e. CORE, cardio-oncology rehabilitation, and exercise) to improve cardiovascular outcomes in cancer patients and survivors. Given the interrelatedness of cancer and CVD,^13^ the widespread adoption of a CORE model could improve both CVD and cancer outcomes in at-risk and affected individuals.

The International Cardio-Oncology Society (ICOS) established a CORE working group (WG) in early 2022 to help address these challenges and to accelerate discovery and knowledge translation in the field. Currently, clinical CORE programmes are not considered standard of care. The lack of third-party reimbursement is arguably one of the largest barriers to CORE widespread adoption. However, several important preceding and complementary factors need to be addressed before universal third-party reimbursement can become widely adopted. These factors include the lack of (i) robust evidence demonstrating the direct impact of this intervention model on CVD morbidity and mortality; (ii) awareness of CORE's potential benefits within the medical community; (iii) evidence, algorithms, and systems to inform and support patient triage and referrals; (iv) curriculum and educational opportunities to train specialized cardio-oncology rehabilitation professionals; and (v) institutional resources (e.g. physical, financial) and infrastructure to support CORE programmes suited to a diverse patient group. The current lack of robust evidence is the most ‘upstream’ of these factors. Thus, improving the rigour and completeness of the CORE evidence base is a critical antecedent to achieving numerous ‘downstream’ priorities in the field. This first paper from ICOS's CORE WG aims to support this critical antecedent step by reviewing and expanding current cardio-oncology practice definitions within the context of CORE, highlighting the findings and limitations of CORE and CORE-related research, proposing research priorities for the field, and recommending standards for CORE research.

*Development*: The development of this whitepaper (including scope and focal points) began during a WG meeting in May 2022. During and following the meeting, the WG 20 members reviewed 3 relevant Delphi studies^21–23^ and openly discussed the need for, scope of, and the best methods to adopt for the project. The WG decided to use non-Delphi, expert consensus methods for the project, given the uniformity of the research priorities and research standards discussed during the preliminary discovery and planning meetings.^24^

### CORE research priorities

The WG members were asked to independently develop a list of preliminary research priorities for the field of CORE while considering the following criteria:

1. Population-level impact; number of patients affected
2. Severity of clinical manifestations or adverse outcomes
3. Feasibility of the proposed research
4. Potential for paradigm disruption or highly novel discovery
5. Translatability (bench to bedside to bench)
6. Multidisciplinary importance

Beginning in May 2023, the CORE research priorities proposed by the WG members were consolidated and, if necessary, combined by the lead authors (S.C.A. and E.H.). The WG asynchronously reviewed and discussed the developing list until a final research priority list was circulated in February 2024. Our WG members continued asynchronously discussing and refining the list until consensus was achieved (early July 2024).

### CORE research standards

Concurrent with developing the CORE research priorities, the WG members were asked to consider and develop a set of CORE research standards. The overarching objective was to identify the most critical aspects of CORE research needed to address current gaps in the evidence base and improve the rigour of CORE research to support downstream priorities in the field. The WG agreed to partition the CORE research standards into ‘Ideal’ vs. ‘Minimum’ criteria to account for the variability in socioeconomic status and the resulting availability of physical and financial resources to support CORE research across countries and regions. Whenever possible, the WG recommended using existing and widely accepted documents [e.g. the Consolidated Standards of Reporting Trials (CONSORT) guidelines] and gold-standard methods (e.g. use of CPET) to support the development and implementation of CORE research. The timing of the initial proposal, consolidation and combination, discussion, and refinement of the final set of CORE research priorities was aligned with the development of the CORE research priorities.

## CORE: the missing link in cardio-oncology care?

**Figure 1:**
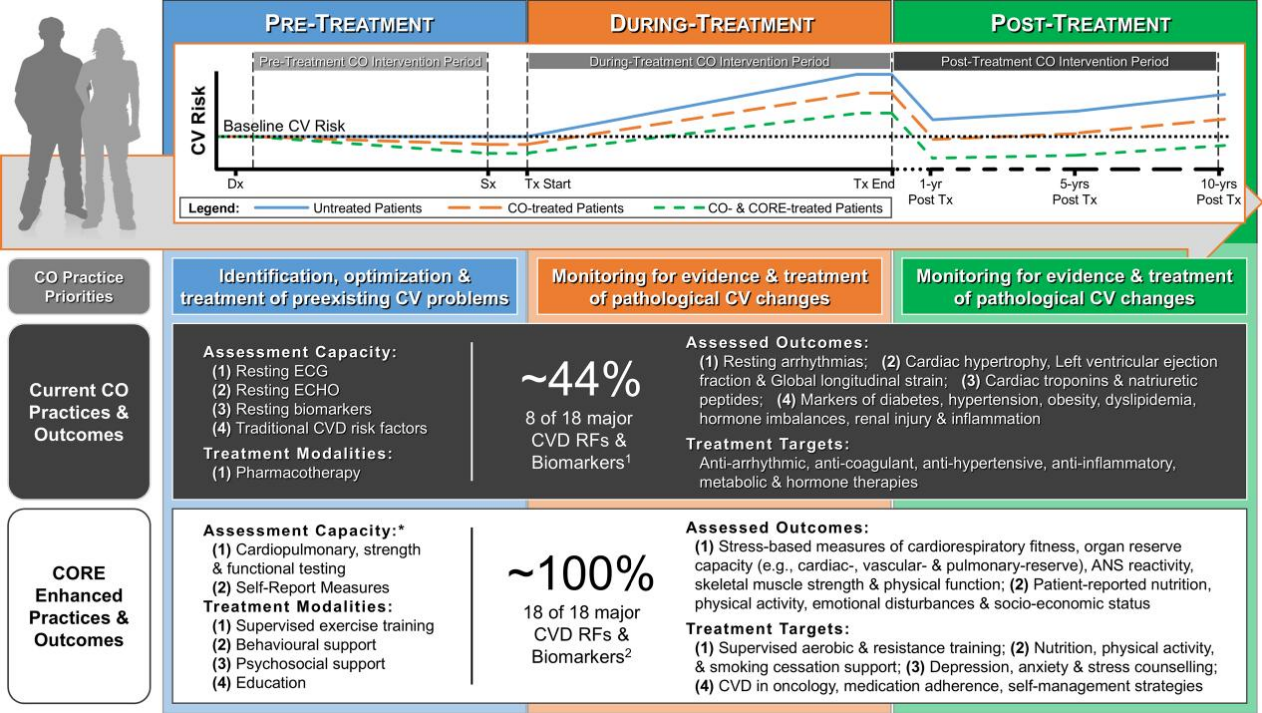
CORE-optimized CO care model. ANS, autonomic nervous system; CO, cardio-oncology; CORE, cardio-oncology rehabilitation and exercise; CV, cardiovascular; CVD, cardiovascular disease; Dx, diagnosis; ECG, electrocardiogram; ECHO, ultrasound-based echocardiography; RF, risk factor; Tx, therapy. *May facilitate earlier detection of *de novo* or worsening subclinical and overt cardiovascular abnormalities. ^1^(1) Resting measures of cardiac structure and function; (2) renal injuries and impairments; (3) obesity; (4) diabetes; (5) dyslipidaemia; (6) hypertension; (7) inflammation; and (8) hormone imbalances. ^2^(1) ANS reactivity; (2) cardiorespiratory fitness; (3) stress-based measures of cardiac, pulmonary, peripheral vascular, and skeletal muscle reserve; (4) nutritional habits; (5) physical activity levels; (6) smoking status; (7) emotional disturbances (e.g. depression, anxiety, and distress); and (8) socio-economic status

The discipline of cardio-oncology has the overarching goal of facilitating the delivery of optimal cancer care while minimizing cancer treatment-related cardiovascular toxicity.^17^ According to the National Cancer Institute, the continuum of cancer care extends years beyond the cessation of cancer treatment,^25^ and long-term care planning is recommended for all survivors who received anti-cancer therapies as they remain at risk for late adverse effects, like CVD.^26,27^ Well-defined roles have been proposed for cardio-oncology teams that span the pre-treatment, during-treatment, and post-treatment phases of the cancer care continuum^17^ (*Figure 1*). However, the current clinical practice of cardio-oncology care across these phases is limited, involving (i) clinical surveillance strategies that are primarily restricted to risk assessment^28^ at baseline (i.e. pre-treatment), routine imaging-based monitoring of resting cardiac function during treatment,^18^ and (ii) pharmacotherapy-based intervention strategies delivered in either primary or secondary prevention settings.^18^ On the other hand, recent evidence suggests that stress-based measures (e.g. those assessed via CPET) provide insight into cardiac, pulmonary, vascular, and skeletal muscle reserve that may offer additional benefit when combined with traditional approaches when characterizing survivors’ cardiovascular health status and CVD risk.^29^ Most cardio-oncology guidelines and position statements endorse promotion of healthy lifestyle and education^17,18^ as critical elements of cardio-oncology care; however, these recommendations are seldom implemented. These intervention elements likely represent a critical missing link between the stated goals of the cardio-oncology discipline (i.e. reducing cardiovascular toxicity and CVD risk in cancer survivors) and our ability to achieve better short- and long-term cardiovascular and cancer outcomes for cancer survivors.

The landmark position statement by the AHA and ACS proposed the adoption of a cardiac rehabilitation care model that is tailored to address the unique cancer- and treatment-related mechanisms of cardiovascular injury and the specific educational and behavioural support needs of this population to improve cardiovascular outcomes in ‘at-risk’ cancer survivors (*Table 1*).^19^ The following sections build upon this work by discussing how integrating CORE into current cardio-oncology practice may help support the strategic objectives of the field, the care objectives of healthcare practitioners (HCPs), and improve outcomes for patients and healthcare systems.

**Table 1:** Important features of AHA / ACS’ cardio-oncology rehabilitation position Stand**^19^**

Key Features	Details
Patient referral algorithm	Decision tree supporting patient referrals to cancer rehabilitation, CORE, and community-based exercise programmes based on patients’ treatment exposure, symptoms / diagnosis, and need for supplementary consultations.
Safety checklist for exercise training	Standard testing (CPET, Graded Exercise test, 6-min walk test, blood work investigations); Symptoms; Ongoing cancer complications; Patients’ exercise knowledge
General and cancer-specific considerations for the key CORE components	(1) Patient assessment; (2) Nutrition counselling; (3) Optimal weight management; (4) Blood pressure management; (5) Lipid / lipoprotein management; (6) Diabetes mellitus management; (7) Tobacco cessation; (8) Psychosocial management; (9) Physical activity counselling; and, (10) Exercise training
Considerations for providing on-site vs. home-based CORE	Key strategies for on-site programmes and recommendations for adapting care to home-based settings

## Cardio-oncology care and research: scope and recent developments

**Figure 2:**
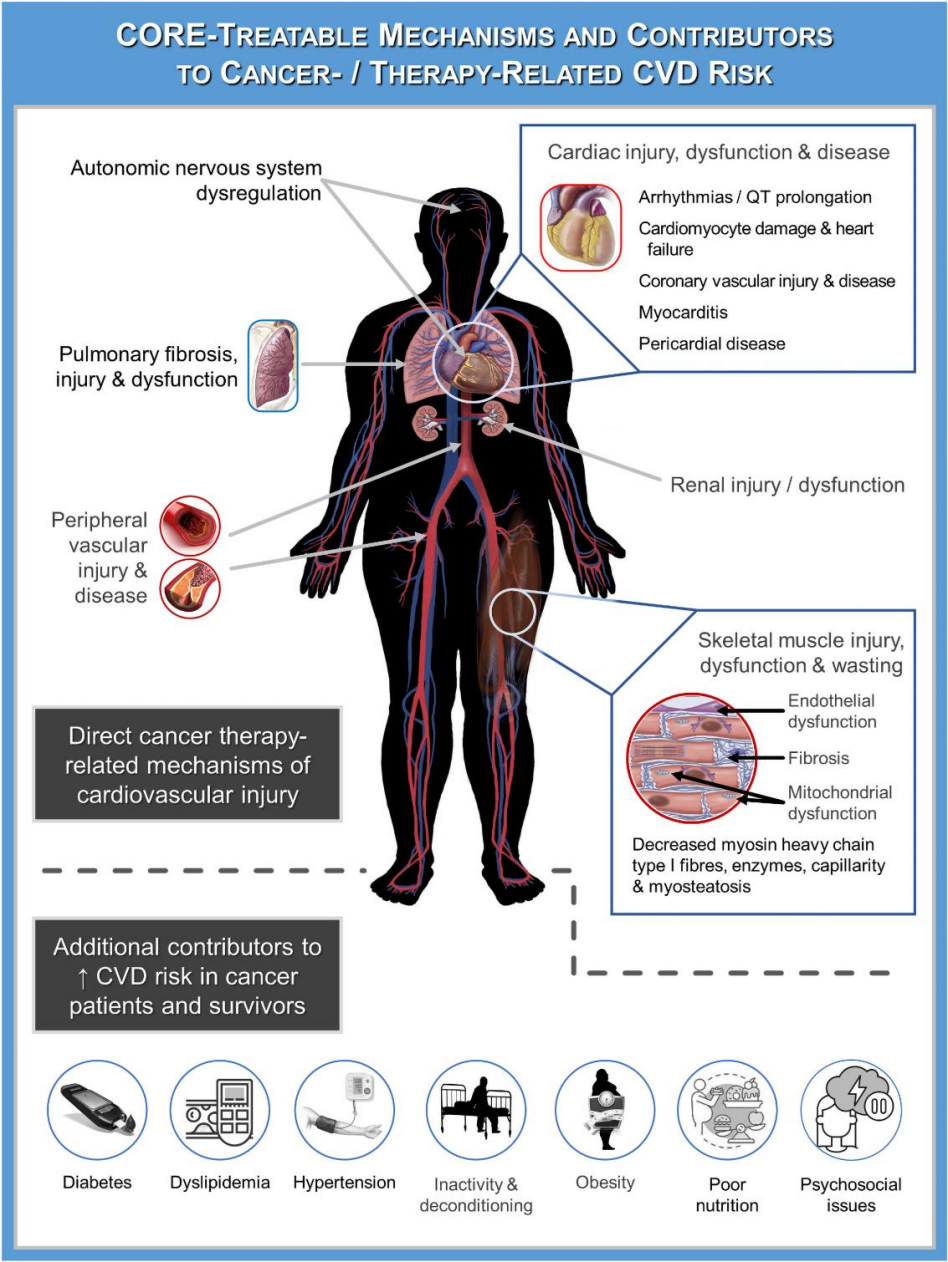
CORE-treatable mechanisms and contributors to cancer therapy-related CVD risk. CVD, cardiovascular disease

Emerging cancer survivorship data indicate that CVD-related mortality often exceeds cancer-related mortality beyond 5–7 years post-diagnosis.^30,31^ There are many adverse cardiovascular effects of cancer and its treatments that contribute to this increased mortality risk. For example, Strongman *et al*. reported that survivors of the 20 most common adult cancers had increased medium- to long-term risk for at least one type of CVD (i.e. coronary artery disease, arrhythmia, heart failure or cardiomyopathy, stroke, peripheral vascular disease, venous thromboembolism, pericarditis, and valvular heart disease) relative to the general population.^32^ These findings are aligned with numerous studies reporting increased CVD risk in specific cancer types, including breast,^30^ germ cell,^33^ prostate,^34^ and hematologic malignancies,^35,36^ as well as mixed cohorts of adolescents and young adult cancer survivors who develop CVD risk factors.^37^ The most well-characterized cardiovascular complication of cancer treatment is cancer therapy-related cardiac dysfunction (CTRCD). These complications were defined in an ICOS consensus statement^38^ and endorsed by the recent European Society of Cardiology guidelines.^18^ They highlight the scope of cardio-oncology practice and research by defining five classes of acute cardiovascular toxicities, including (i) cardiac dysfunction/heart failure (e.g. CTRCD), (ii) arrhythmias/T prolongation, (iii) myocarditis, (iv) acute hypertension, and (v) vascular toxicity. This expanded classification system reflects important advances in the field by incorporating additional and more common^39^ markers of cardiac injury/dysfunction and select cardiovascular sequelae beyond the heart (e.g. hypertension, peripheral vascular disease). However, even this expanded classification system does not completely capture the breadth of acute and short-term cancer treatment-related sequelae of the extended cardiorespiratory system (e.g. injuries to the lungs,^40^ nervous system,^41,42^ and skeletal muscles^43–46^) or consider other likely contributors to near- and long-term CVD risks (e.g. physical impairments and frailty,^47^ emotional disturbances,^48^ CVD risk factors^39^) in cancer survivors (*Figure 2*). Moreover, ongoing progress in medical oncology has led to the introduction of novel therapeutics with different forms of cardiovascular consequences—making it difficult to risk stratify patients receiving cutting-edge therapies effectively.

Indeed, routine clinical monitoring may fail to detect sub-clinical, physical, and psychosocial sequelae of cancer therapy. Consequently, overt CVD may only become problematic years into survivorship due to the long-term cardiovascular consequences of cancer therapies and underlying comorbidities. A retrospective study from Abdel-Qadir *et al*.^39^ found that early-stage breast survivors without pre-existing CVD risk factors were rarely admitted to hospital (<3% of cases) for heart failure-related reasons. There is a great need and opportunity to reduce the individual and societal burden of cancer-related CVD by augmenting risk factor management beyond traditional pharmacotherapy-based approaches. Ultimately, minimizing the overall burden of cancer-related CVD requires establishing comprehensive care strategies for cancer patients and survivors with elevated CVD risk that involve personalized CORE-based treatment of the specific mechanisms of CVD injury or risk identified via rigorous screening and follow-up practices.

## CORE research: definitions, evidence, and knowledge gaps

### CORE vs. CORE-related research

AHA/ACS's CORE position provides a summary of evidence demonstrating the effects of exercise therapy on cardiovascular outcomes during and following treatment in cancer survivors. The review highlighted randomized studies reporting exercise-mediated effects in cardiorespiratory fitness,^49–60^ vascular function,^49,53^ cardiac function,^61^ CVD risk factors,^49,53^ and observational evidence on CVD events,^52,62,63^ CVD-related mortality,^62^ and all-cause mortality^63,64^ in mixed groups of patients and survivors, including breast, prostate, colorectal, testicular, and haematological malignancies. While encouraging, much of this evidence is considered indirect, as many studies were not specifically designed to address cardiovascular sequelae in at-risk or affected populations. Progress in the field of CORE will depend on the specificity, quality, and demonstrated efficacy of future research. Thus, for the purpose of this review, we define and distinguish between direct ‘CORE research’ and ‘CORE-relevant research’ as studies at least meeting the first, second (a/b), and fourth PICO-defined criteria as defined below:

1. *Patient/population*: randomized controlled trials (RCTs) intentionally recruiting participants based on living with or having an increased risk of cancer treatment-related cardiovascular dysfunction, CVD, or related mortality.
2. *Intervention (CORE research)*: RCTs testing multi-component intervention strategies (i.e. exercise therapy, physical therapy, physical activity, nutrition therapy, pharmacotherapy, education, behavioural support) specifically designed to target mechanisms or influence behaviours that may prevent, treat, or improve cardiovascular dysfunction/injury, CVD, or related mortality.
3. *Intervention (CORE-relevant research)*: RCTs testing single-component intervention strategies (i.e. exercise therapy, physical activity, nutrition therapy, pharmacotherapy, education, behavioural support) specifically designed to target mechanisms or influence behaviours that may prevent, treat, or improve cardiovascular dysfunction/injury, CVD, or related mortality.
4. *Comparison*: RCTs involving a control group/condition that should not impact (efficacy designs) or have a lesser impact (superiority designs) on the primary and key secondary cardiovascular outcomes.
5. *Outcomes*: RCTs specifically designed and statistically powered to detect clinically meaningful changes in a clearly defined cardiovascular primary outcome and key secondary outcomes.

According to these standards, none of the 15 studies featured in the AHA/ACS review would be classified as ‘CORE research’ and only 6^49,52,53,56,61,62^ of the 15 studies would be considered ‘CORE-relevant research’ (i.e. meeting PICO criteria 1, 2b, and 4). Thus, these studies provide limited insight into the full potential of the multi-component CORE intervention model. To address this knowledge gap, Fakhraei *et al*.^65^ conducted a systematic review and meta-analysis of 10 studies that reported testing cardiac rehabilitation-based intervention models in cancer survivors. The meta-analysis findings suggested that completion of cardiac rehabilitation-based interventions was associated with more favourable levels of cardiorespiratory fitness and fatigue at the post-intervention assessment. However, the authors reported moderate-to-high levels of risk of bias and low-to-moderate reporting quality across the included studies, which undermines the rigour, validity, and reproducibility of this evidence.^65^ Notably, despite claiming to test cardiac rehabilitation-based intervention models, none of the included studies adequately reported on the inclusion, details, or delivery of non-exercise-based intervention components. Moreover, none of the prospective studies included in the review^65^ meet the criteria for being ‘CORE research’ given that they did not intentionally target populations with known or increased rates or risk of cancer-related CVD. A related meta-analysis, including many of the same studies, reported that exercise-based multimodal interventions significantly improved multiple physical and psychological health indices in these patients.^66^ However, we agree with Fakhraei and colleagues, who emphasized caution against making causal attributions based on this evidence, given that most studies employed non-specific recruitment strategies and were poorly described, single-arm/non-randomized trials with high attrition rates.^65^ Based on the narrow/non-specific scope (e.g. single modality interventions, involvement of low-risk/unaffected patients, non-cardiovascular primary outcomes) or poor quality of the available evidence, there remains uncertainty regarding how the co-existence of cancer- and CVD-related factors within cardio-oncology survivors impacts the safety, tolerance of, and acute responsiveness to CORE interventions, as well as the long-term health benefits.

## Current evidence from CORE-related research

Since the publication of the aforementioned systematic reviews, there have been several CORE RCTs published. The TITAN trial tested the efficacy of a multidisciplinary cardiac rehabilitation intervention that included cardiovascular risk factor management, exercise, and nutrition components vs. usual care in women with early-stage breast cancer receiving either trastuzumab or anthracycline chemotherapy.^67^ The intervention was delivered over a 12-month period and found no difference in the primary outcome of changes in resting left ventricular ejection fraction (LVEF) at follow-up. The ONCORE trial, which also included early-stage breast cancer survivors receiving either anthracycline chemotherapy or trastuzumab, evaluated an exercise-based CORE programme plus standard of care vs. standard of care, which included monitoring for cardiotoxicity and management of cardiovascular risk factors.^68^ The intervention attenuated the reductions in resting LVEF and increases in body mass that were observed with standard of care. The incidence of cardiotoxicity (defined as a reduction in LVEF of <10% from baseline) was low in both studies (i.e. 4% in TITAN and 8% in ONCORE). Finally, the CORE study, which recruited participants with higher cardiovascular risk (according to the American Society of Clinical Oncology Practice Guideline^26^) tested a 2-month multi-modal intervention of exercise training, dietary advice, and cardiovascular risk factor management on cardiorespiratory fitness and cardiovascular risk factors.^69^ The intervention caused significant improvements in peak oxygen uptake, cardiovascular risk factor control, quality of life, and health literacy,^70^ and was shown to be cost-effective in managing cardiovascular risk within high-risk cancer survivors.^71^ Collectively, these studies provide preliminary evidence that CORE-based interventions are safe and well tolerated (i.e. no adverse events reported in any of the studies) and that multi-modal interventions with a minimum duration of 2 months may be effective at improving cardiovascular health in cancer survivors. However, there was also evidence that longer durations of CORE-based interventions may be vulnerable to issues with adherence and may, therefore, benefit from including other supportive care components to maintain engagement to maximize effectiveness.

The results of related trials that meet the PICO-defined criteria for being ‘CORE-relevant research’ support the findings of these early CORE studies. We conducted a comprehensive review of definitive RCTs, smaller RCTs, and non-randomized trials evaluating the efficacy of CORE and CORE-related care models in an oncology setting. The following sections describe the evidence and knowledge gaps according to the findings of these CORE-relevant studies assessing the effects of key CORE intervention components, including (i) exercise, (ii) diet, (iii) psychosocial management, (iv) risk factor management, and (v) physical activity counselling on cardiovascular health and risk factors.

### Exercise intervention effects

Systematic reviews and meta-analyses of pre-clinical studies consistently report that exercise training improves resting cardiac function during and following chemotherapy treatment.^72,73^ On this basis, a growing number of clinical studies have tested the effects of exercise training on markers of cardiac structure and function; however, these studies have reported mixed results. Pooled data from a 2021 systematic review and meta-analysis of four RCTs in breast cancer survivors reported no significant effect of exercise training on resting LVEF [mean difference (MD) 2.07%; 95% confidence interval (CI): −0.17 to 4.34].^74^ Secondary analyses of the 3 studies that tested interventions with ≥36 exercise sessions demonstrated that exercise training prevented the decline in resting LVEF compared with usual care (MD 3.25%; 95% CI: 1.20 to 5.31).^74^ These findings suggest that lower dosage interventions may not have imposed enough total stress on the heart to elicit favourable physiologic (e.g. upregulation of antioxidant defence mechanisms) and functional cardiac adaptations.

Two of the recent CORE-multimodal studies^67,68^ included exercise programmes of ∼5 to 12-months evaluated cardiac effects among women receiving treatment for breast cancer. The exercise interventions were similar in these trials (i.e. twice-weekly sessions of moderate-intensity aerobic exercise and resistance training); only the trial by Diaz-Balboa *et al*.^68^ was found to be cardioprotective by attenuating small reductions in LVEF observed at follow-up. In a study conducted by Foulkes *et al*.^29^ 104 women scheduled to receive anthracycline-based treatment for early-stage breast cancer were randomized to either 12 months of aerobic and resistance exercise or usual care. The intervention involved 3–4×/weekly aerobic exercise sessions (i.e. 30–60 min of endurance training, 35 min of tempo training, and 30–35 min of high-intensity interval training) and moderate-to-high intensity resistance exercise training (i.e. 2 sets of 8–15 repetitions at 60%–85% 1 repetition maximum) during adjuvant/neoadjuvant treatment. At the end of the study, exercise attenuated functional disability (primary outcome) and cardiac troponin levels immediately following cancer treatment and improved cardiorespiratory fitness and cardiac function at peak exercise.^29^ However, the intervention had no impact on resting measures of cardiac function, possibly due to the recruitment of participants with normal resting cardiac function^29^ or that resting measures of cardiac function may lack sensitivity to the therapeutic effects of exercise training on the cardiovascular system—suggesting the potential need to focus on more sensitive, and perhaps more clinically relevant outcomes in the setting of a preserved ejection fraction. For instance, older survivors of breast cancer may have a greater risk of developing heart failure with preserved, rather than reduced, ejection fraction.^75^ These individuals do not present with impaired resting cardiac function but demonstrate a reduced capacity to augment cardiac function during exercise,^75^ similar to people with heart failure with preserved ejection fraction. In addition, a recent study from Dillon *et al*.^76^ demonstrated that a 4-month targeted exercise (2 days/week of 20–30 min of moderate aerobic and 20 min of moderate-to-high intensity resistance training, 1 day/week of 30–35 min of high-intensity interval training) and physical activity (targeting ≥30 min/day reduction in sedentary time) approach elicited similar effects preserving cardiorespiratory fitness and exercise cardiac function in patients with haematologic cancer undergoing allogeneic stem cell transplant. Overall, the findings of Foulkes *et al*. and Dillon *et al*. provide the first rigorous evidence that well-designed, targeted exercise interventions may be capable of attenuating declines in cardiac reserve but not resting systolic function.

Several systematic reviews and meta-analyses of exercise trials in oncology have assessed the effects of exercise therapy on traditional CVD risk factors, finding favourable effects on blood pressure,^77,78^ HDL,^77^ triglycerides,^77^ fasting glucose,^78^ body mass index,^77,79^ and body fat.^78,80–83^ At least thirteen ‘CORE-relevant’ RCTs and non-randomized intervention studies have investigated or explored the impact of exercise training on traditional CVD risk factors in survivors of breast,^84–91^ testicular,^49^ prostate,^53,92^ female,^93^ and mixed cancers.^94^ However, the findings across studies were mixed, with exercise training reported to cause, or be associated with, positive changes in levels of resting blood pressure in 3 of 10 studies,^49,85,89^ resting heart rate in 2 of 6 studies,^49,90^ lipid profiles in 4 of 6 studies,^49,91,92,94^ metabolic blood markers in 3 of 7 studies,^85,93,95^ body habitus in 3 of 5 studies,^85, 90,94^ adiposity in 1 of 4 studies,^85^ and non-trial physical activity engagement in 1 of 4 studies.^93^ Several factors may account for the heterogeneity in these findings. First, exercise trials are prone to sampling bias as participants who are typically healthier or have fewer barriers to participation than the average survivor are more likely to enrol, which limits generalizability. This sampling bias could limit the potential to demonstrate intervention effects (e.g. loss of adipose tissue) within studies as participants with lower CVD risk factors (e.g. less adipose tissue) have less capacity to improve than those who do not. Second, only 2 of the 13 listed studies were designed and statistically powered to detect the effects of exercise on a traditional CVD risk factor as their primary outcome. Third, the included studies tested interventions that were very heterogeneous in terms of exercise modalities (i.e. aerobic exercise-only interventions vs. multimodal interventions involving aerobic, resistance, and behavioural support components) and exercise training frequencies [i.e. ranging from very low (four sessions^90^ in 12–16 weeks—average of 0.25–0.33 sessions per week) to high (208 sessions^29^ in 52 weeks—average of 4 sessions per week)]. Thus, the high degree of intervention heterogeneity makes it difficult to compare and synthesize the data, and more studies are required to determine the most effective type of exercise programme to improve cardiovascular risk factors among cancer survivors.

Other recent systematic reviews and meta-analyses of exercise oncology trials provide evidence that exercise training positively impacts cardiorespiratory fitness,^56,60,96,97^ vascular function,^98^ metabolic factors,^79,99^ skeletal muscle mass^78,79,82,83,100^ and skeletal muscle strength,^60,79,82,83,97,101–103^ inflammatory factors,^78,104,105^ and psychosocial issues.^79,101,106^ In addition to the recent ‘CORE’ study which demonstrated an improvement in peak oxygen uptake,^70^ eighteen ‘CORE-relevant’ RCTs and non-randomized intervention studies have evaluated the effects or associations of exercise-based interventions on these factors, demonstrating that exercise training has favourable effects or associations (i.e. improving, preventing or mitigating injury/dysfunction) on cardiorespiratory fitness in 15 of 17 studies,^29,49,53,76,84,86–88,92–95,107–109^ vascular structure and function in 3 of 3 studies,^49,53,88^ skeletal muscle mass in 2 of 4 studies,^85,92^ skeletal muscle strength in 2 of 2 studies,^88,92^ autonomic nervous system function/reactivity in 3 of 3 studies,^49,84,93^ biomarkers of cardiac injury in 2 of 4 studies,^29,95^ inflammation in 2 of 3 studies,^49,85^ and estimated CVD risk or metabolic syndrome scores in 3 of 3 reports from 2 RCTs.^49,85,91^ There is also emerging evidence that skeletal muscle quality is associated with impaired cardiovascular function in cancer survivors^110^ and could mediate the increased risk of CVD.^46^ Whether exercise or CORE interventions can attenuate these relationships requires investigations. The consistency of findings across these studies is encouraging, supporting the growing belief that exercise may have an important role in improving myriad CVD outcomes in cancer survivors.

### Dietary intervention effects

Recent CORE-related intervention studies incorporated individualised nutritional interventions that were either tailored to meet dietary goals to target improvements in cardiovascular risk factors or based on personalised recommendations designed by an oncology-specialised dietitian. Both interventions demonstrated improvements in cardiovascular risk factors with improvements in systolic blood pressure, and levels of total cholesterol, triglycerides, and low-density lipoprotein^67,70^ and reductions in weight or body mass index, lean and fat body mass, and waist and hip circumference.^70^ These studies provide the first preliminary evidence that targeted dietary interventions can improve cardiovascular outcomes for cancer survivors with increased CVD risk.

There is currently insufficient evidence for or against dietary interventions such as ketogenic or low-carbohydrate diets, low-fat diets, functional foods, or fasting to improve outcomes related to quality of life, treatment toxicity, or cancer control.^111^ However, the cardiac rehabilitation framework incorporates nutritional support and the recommendation for a healthy diet as a cornerstone of CVD prevention and management for all individuals.^112,113^ The major international cardiac rehabilitation and cardiovascular societies (e.g. AHA, European Society of Cardiology) recommend the adoption of the Mediterranean diet or similar diets (e.g. DASH); limiting saturated fat intake, reducing salt intake, and eating more plant-based foods and fibre-rich foods, including whole grains, fruits, vegetables, and nuts; and minimizing intake of foods and beverages with added sugar to lower the risk of CVD. However, direct evidence supporting the efficacy of these approaches and recommendations specific to cancer survivors is lacking. A Cochrane review found that randomized studies evaluating the effects of dietary interventions for cancer survivors had little effect on overall mortality or secondary cancers.^114^ Although a more recent systematic review suggested that a Mediterranean diet was associated with a lower risk of mortality in prostate cancer survivors, better diet quality was associated with improved survival in breast and colorectal cancer survivors, and generally when dietary interventions are combined with physical activity there are benefits to quality of life.^115^ There is emerging evidence that certain diets may impact cancer and CVD risk with mechanistic data suggesting that higher intake of animal-based foods (especially those that are highly processed) can promote inflammation, oxidative stress, gut dysbiosis, endothelial damage, and excess adiposity through the consumption of saturated fat, Neu5GC-glycans, trimethylamine N-oxide, heme iron, advanced glycation end products, and heterocyclic amines.^116^ Conversely, predominantly whole-food plant-based nutrition promotes healthy weight, improves insulin sensitivity and vascular health, and decreases inflammation and oxidative stress.^117^ For example, the LEAN trial evaluated a counselling programme that focused on caloric reduction, predominantly plant-based diet, mindful eating practices, and physical activity in breast cancer survivors, finding a 30% reduction in C-reactive protein levels and >5% weight loss after 6 months in the intervention groups.^118^

### Multi-modal CVD risk factor management intervention effects

Education on health risks and behaviours, as well as pharmacotherapy-based CVD risk factor management, are central components of the CORE care model. These strategies have been demonstrated to effectively reduce the frequency of recurrent adverse events in patients who have had a myocardial infarction^119^ and heart failure.^120^ Preliminary evidence from the findings from the CORE trial and TITAN trial suggests that these approaches may be effective when tailored to cardiovascular risk factors.^67,70^ Engaging patients in goal setting and action planning to foster enhanced patient activation and disease self-management is recommended in cardiac rehabilitation,^121^ though these approaches require further investigation in the CORE setting. To efficiently reduce levels of traditional CVD risk factors, most patients will require intensive health behaviour interventions and pharmacological therapies. The development of individual, patient-centred plans should focus on optimizing risk factors according to clinical practice guidelines appropriate for each risk factor. Hypertension is the foremost modifiable risk factor with adverse cardiovascular outcomes among oncology patients.^122^ To date, there are no cancer-specific blood pressure targets; thus, we recommend CORE programmes follow local and international guidelines (ACC/AHA or ESC guidelines),^123–125^ with the caveat that there are limited data supporting suggested thresholds as cancer patients were excluded from RCTs. CORE programmes should encourage non-pharmacological interventions to reduce blood pressure, such as weight loss, heart-healthy dietary patterns, sodium reduction, dietary potassium supplementation, and limiting alcohol consumption.^123^ CORE programmes should also address other pertinent CVD risk factors like smoking cessation (including vaping). Smoking cessation in cancer survivors is associated with improved survival, with earlier intervention being associated with the greatest benefit.^126^ CORE-specific research in this area is needed to establish and validate CVD risk factor targets and, ultimately, management guidelines.

### Psychosocial intervention effects

Psychosocial interventions for people with cancer generally improve depression and quality of life.^70,127–129^ However, more data are needed to propose a standardized framework for informing the nature and extent of the psychosocial support required to treat individual patients. A recent trial demonstrated a multi-modal intervention that included a psychological management component to address psychosocial concerns and enhance motivation for adopting health behaviours,^68^ improved quality of life, anxiety, and depression in survivors with high CVD risk.^70^

Stress affects multiple systems within the body, including the sympathetic nervous system,^130^ cardiovascular^131^ and immune function,^132^ and hypothalamic-pituitary-adrenal axis,^133^ which could worsen health outcomes for cancer survivors. Thus, modifying responses to common stressors in cancer patients and survivors may also improve quality of life, cardiovascular, and health outcomes. Stress management intervention (SMI) strategies are recommended components of cardiac rehabilitation programmes. Several systematic reviews and meta-analyses have tested and shown the benefits of SMIs in an oncology setting.^134–137^ However, most recent CORE trials have yet to implement SMIs; thus, to our knowledge, there is no evidence to inform the implementation of SMIs within CORE settings.

### Behaviour change intervention effects

Helping patients adopt protective health behaviours is an important goal of cardiac rehabilitation programmes. There is a large amount of evidence supporting the benefits of behavioural counselling and behavioural therapies in the oncology setting. For example, several recent systematic reviews have reported that behaviour change interventions improve important CVD-related outcomes like moderate-to-vigorous physical activity participation, body mass index, and fatigue and may support improvements in other outcomes like sleep quality.^138–140^ However, the effects of behaviour change studies targeting improvements in dietary outcomes are inconsistent.^141^ The potential downstream benefits of successful behaviour change interventions are well documented, with well-established evidence from meta-analyses and RCTs that regular physical activity engagement is associated with better symptom and morbidity profiles, lower cancer recurrence risk, and reduced mortality risks (e.g. all-cause, cancer-specific) in cancer survivors. Notably, these relationships appear to be consistent between the general exercise oncology evidence-based and ‘CORE-relevant’ cohort studies conducted to date. Specifically, five ‘CORE-relevant’ observational studies published between 2014 and 2021 included data from samples ranging between 1187 and 39 775 survivors of breast cancers,^52,142,143^ Hodgkin lymphoma,^144^ and mixed paediatric cancers^63^ have demonstrated that higher levels of physical activity engagement in the pre- and post-treatment settings, particularly vigorous intensity physical activity, is associated with reduced risks of CVD events,^52,143,144^ CVD incidence,^142^ and all-cause,^63^ CVD-specific^143^ and health-related mortality.^63^ Scott *et al*.^63^ further assessed the associations between changes in vigorous-intensity physical activity behaviour over an 8-year period, finding survivors who increased and maintained higher levels of vigorous-intensity physical activity had significantly lower risk of all-cause mortality compared with survivors who maintained low levels of vigorous-intensity physical activity over the same period. There is emerging evidence that the assessment of cardiorespiratory fitness may help risk-stratify survivors according to long-term mortality risk. A seminal study by Groarke and colleagues^145^ evaluated the associations between low, moderate, and high levels of cardiorespiratory fitness and risks of all-cause, CVD-related, and cancer-related mortality in a mixed cohort of 1632 survivors an average of seven years post-diagnosis. Compared with survivors with low fitness, all-cause and cancer-related mortality risk was significantly lower in survivors with moderate fitness (HR 0.38; 95% CI: 0.28–0.52) and lowest in survivors with high fitness levels (HR 0.17; 95% CI: 0.11–0.27), with a similar significant reduction in CVD-related mortality in survivors with moderate (HR 0.40; 95% CI: 0.19–0.86) and high fitness levels (HR 0.41; 95% CI: 0.16–1.05).^145^ Moreover, the authors reported a 14% to 26% reduced risk of all-cause, CVD- and cancer-specific mortality per 1 MET increase in cardiorespiratory fitness.^145^ Collectively, these findings provide compelling preliminary evidence that physical activity behaviour and cardiorespiratory fitness may be important CVD-related screening and risk assessment targets and intervention targets for CORE programmes and CORE-related support services.

## Current CORE research activities and priorities

Against this background, there is a clear need for rigorously designed and conducted studies that are adequately powered to test the effects of CORE-based interventions on established and emerging CVD risk factors and biomarkers within well-defined groups of patients/survivors living with or at risk of cancer-related CVD. While related and complementary investigations are needed to advance this field, we recognize there are challenges inherent to exercise and CORE-related research that remain unresolved, including specific biases (e.g. lack of patient and investigator blinding), intervention reproducibility and reporting, and variable access to financial and human resources between centres and countries.

The ICOS CORE WG aims to accelerate advances and reduce unnecessary redundancy in this field in several important ways. First, our group has proposed a list of research priorities to complement the work of ongoing trials and address the major and most proximal knowledge gaps in the field (*Table 2*). Second, our working group is hosting a CORE research platform (https://ic-os.org/committees/exercise/). The primary objectives of the repository are to reduce research redundancy and improve international awareness, coordination, collaboration, and trainee development across similar research programmes by providing investigators/stakeholders the opportunity to promote their work, collaboration interests, and trainee development opportunities within a single, open-access website. A major benefit of a well-curated and -used repository is that investigators will be able to quickly identify the current outcomes being targeted, intervention approaches used, and target populations. Third, this WG has developed standards for CORE research conduct and reporting that are based on best practices within related fields and expert consensus that distinguish between ideal vs. minimum standards. These standards aim to facilitate the conduct of RCTs and observational trials that can provide the rigorous evidence needed to most efficiently address the current knowledge gaps in the field and support the translation of this evidence into practice (see next section).

**Table 2:** Key CORE research priorities

Research phases and types	Research priorities
Phase I (CORE and CORE-related research)	Exploratory studies to identify patient/survivor groups and sub-groups with elevated risks of CV injury, CVD, and risk factors thereof; mechanistic studies to identify the common and unique causes of CV injury and increased CVD risk in individual patient/survivor groups and sub-groups; exploratory studies to characterize the group-specific responsiveness, benefits, and risks of single modality and multimodal intervention strategies on CORE-related outcomes; mechanistic studies to characterize the effects of targeted interventions on the oxygen cascade components and other critical organs and organ systems within patient (sub-) groups; exploratory studies determining the pre-diagnosis and acquired predictors of CV injury, CVD risk, and intervention responsiveness, benefits, and risks within these (sub-)groups; and qualitative and/or phenomenological studies evaluating the ‘lived experience’ of CORE participants, including the preferences, barriers, and facilitators to intervention initiation and maintenance.
Phase II (CORE and CORE-related research)	The efficacy and safety of single- (CORE-related) and multimodal CORE interventions on important CORE-related outcomes in high-priority patient/survivor (sub-)groups; how single and interactions between disease, treatment, and individual characteristics impact CORE and CORE-related intervention adherence and sustainability, the determinants thereof; general, group, and group-specific strategies to improve CORE and CORE-related intervention safety, efficacy, tolerability, adherence, and sustainability; and approaches to targeting CORE-related and CORE interventions to address the unique causes of CV risk and CVD overall and within specific (sub-)groups.
Phase III (CORE research)	The effectiveness, efficacy and safety of multimodal CORE interventions on important CORE-related outcomes in high-priority patient/survivor (sub-)groups; the clinical relevance of the intervention effects; the minimally effective and optimal timing, type, frequency, volume, and combination of intervention components to improve both critical and ancillary CORE-relevant outcomes within and between high priority patient (sub-)groups; whether CORE intervention strategies can positively impact clinically relevant endpoints, like the incidence rates of CVD risk factor development, CVD, unplanned CVD-related hospitalizations, and mortality; if CORE interventions can improve oncology care and related outcomes, like treatment interruption and completion rates; and the cost-effectiveness of CORE interventions via direct implementation research.

## CORE research standards and recommendations

**Figure 3:**
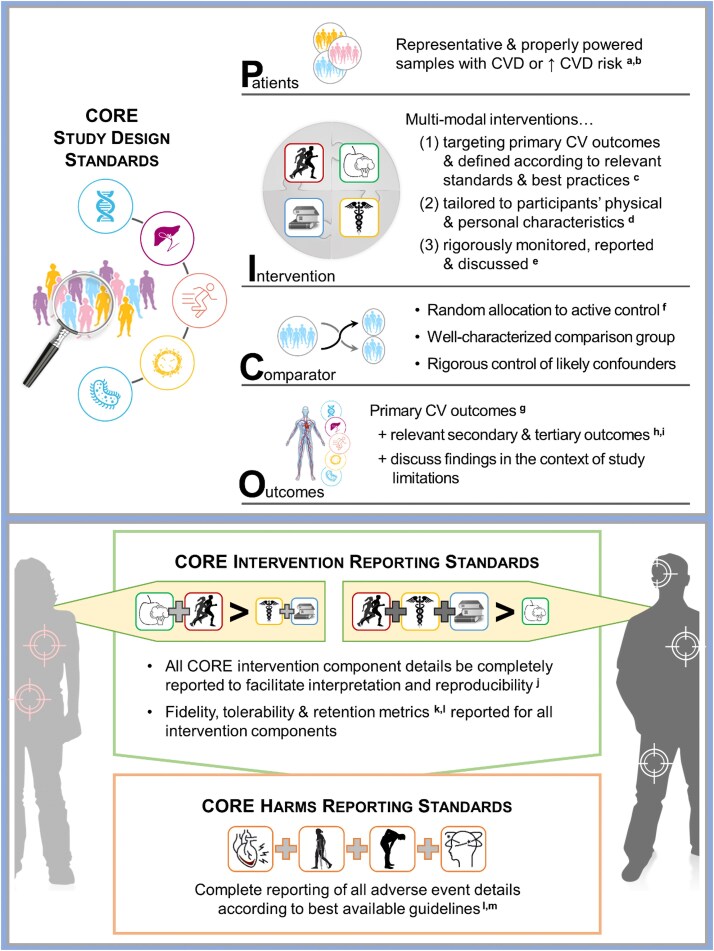
CORE research standards. CV, cardiovascular; CVD, cardiovascular disease; CORE, cardio-oncology rehabilitation and exercise. ^a^May require extended duration and multi-site recruitment strategies when targeting smaller populations. ^b^Participants’ disease, treatment exposures, and relevant medical histories must be well characterized to facilitate interpretation. ^c^According to FITT-P (frequency, intensity, time, type—progression) or best available criteria (non-exercise interventions) to support complete reproducibility for all intervention components. ^d^ Based on ‘gold standard’ (e.g. cardiopulmonary exercise testing) or the most accurate and reliable methods available, outcomes of baseline assessments, comorbidities and health status, and relevant personal characteristics (e.g. socio-economic status, proximity to care facilities). ^e^In the context of the intervention's limitations (e.g. method of tailoring [e.g. peak oxygen consumption vs. % heart rate vs. rating of perceived exertion]; fidelity [e.g. adherence, dose modifications, interruptions]). ^f^Minimum suggested standard is a minimally active/supported control group (e.g. self-directed and unsupported physical activity group; supervised stretching; behavioural support). ^g^Outcomes directly reflective of CV system function and health as well as those implicated in the pathogenesis of CVD. ^h^Outcomes with direct relevance to improving cardiovascular outcomes (e.g. increasing physical activity or intake of omega-3 fatty acids, treating depression, supporting smoking cessation) AND key explanatory variables that influence the study's main outcomes of interest. ^i^To inform the conduct of properly powered randomized controlled trials, underpowered pilot/feasibility studies should primarily focus on rigorously assessing and reporting feasibility and fidelity outcomes (rather than efficacy) but tailor studies to reflect the design of the subsequent randomized controlled trials (e.g. tailoring CORE intervention components to target a primary cardiovascular outcome). ^j^According to their FITT-P (frequency, intensity, time, type—progression) and CERT (consensus on exercise reporting template) guidelines for exercise components or best available methods for non-exercise intervention components, as appropriate. ^k^Such as intervention adherence, dose modification, treatment discontinuation. ^l^See Nielsen *et al*.^146^ for example best practices in intervention reporting and apply these practices to rigorously reporting on all CORE intervention components, as appropriate. ^m^Such as Exercise Harms Reporting Method (ExHaRM) or Consolidated Standards of Reporting Trials (CONSORT)-Harms extension.

Research standards provide practical and theoretical guidance to optimize the rigour, interpretation, and reproducibility of evidence within and across scientific disciplines. Consequently, the WG set out to develop a set of CORE research standards (see ‘Whitepaper Background and Development’ section for the description of methods). This section highlights the existing guidelines CORE researchers can use to inform all aspects of their research (i.e. design to dissemination) and discusses key CORE-specific research considerations. The intention of this guidance is not to obstruct the conduct of clinical trials; rather, we hope to inform CORE-related research practices to enhance the quality of the evidence-based and, therein, accelerate clinical translation. CORE is a relatively nascent field of research. ‘Therefore, this preliminary set of recommendations will primarily focus on considerations that support earlier phases of discovery (i.e. research Phase I and II)’. We also recognize that implementing some recommendations may be challenging due to practical constraints (e.g. physical, human, and financial resources) and local barriers to conducting clinical trials. Thus, based on expert consensus within our working group, we have framed our recommendations according to ‘ideal’ vs. ‘minimum’ standards for CORE research (*Figure 3*).

### General standards for CORE trial design, conduct, and reporting

Several widely available general guidance documents can be used to inform planning, implementation, and reporting for different types of health research (e.g. EQUATOR Network). More recently, the Good Clinical Trials Collaborative published a set of guidance criteria exclusively for clinical trials^147^ that have been updated for contemporary research settings and have been endorsed by international cardiology associations^148^ and should be adopted in research practice. The fundamental elements of CORE studies should be defined according to the PICO criteria, with their conduct and reporting supported by widely available guidelines and best-practice recommendations. Readers are directed to *Table 3* for a summary and the *Online*  Supplementary data for a complete discussion of the proposed ideal and minimum recommendations for the critical aspects of CORE research.

**Table 3:** Ideal and minimum standards for CORE research

Priority areas	Ideal vs. minimum standards and details	
CORE study design		
Patients/ population	Ideal:	Well-defined groups with high CVD risk, established CVD or cardiac toxicities we recommend using multicenter study designs to achieve adequate sample size
	Minimum:	Rigorous pilot/feasibility studies documenting key participant characteristics required to interpret and reproduce studies, including: specific cancer diagnoses; specific doses and scheduling of anti-cancer therapies; and, relevant medical characteristics (e.g. traditional and novel CVD risk factors, biomarkers)
Interventions (general)	Ideal:	General interventions: Specifically designed to target improvements in cardiovascular, metabolic, and psychosocial health and function, quality of life, and survival outcomes Exercise interventions: Rigorous designed and defined according to the primary principles of exercise prescription (FITT-P; frequency, intensity, time, type and progression) Personalized and progressed for each participant using data derived from baseline or interim CPET (e.g. % of achieved VO_2_peak or peak power output) and 1–10 RM (e.g. % maximum /exercise) Tailored delivery formats (e.g. virtual, in-person) to optimize feasibility and accessibility without compromising study and intervention rigour Co-interventions: Nutritional, psychosocial, and behavioural education should be tailored and individualized Pharmacological adherence based on the local and international guidelines
	Minimum:	Investigators should personalize all CORE intervention components to the best of their abilities based on available resources, reporting all relevant methods details and their relative strengths and limitations Reporting critical elements of the intervention such as intensity, attendance, compliance, adherence, interruption, adjustments and discontinuations^141^ Collect and prepare to report the details required to replicate and implement all components of the tested CORE intervention and, where possible, provide evidence of fidelity (e.g. attendance, adherence, tolerability) Document and prepare to report clear theoretical rationale and practical intervention descriptions of how each component is appropriately dosed and tailored
Comparisons	Ideal:	Intervention studies should be randomized and adhere to clinical trial best practices if feasible
	Minimum:	Anticipate, control for (by study design), and collect data on critical intervention cofounders (e.g. non-trial physical activity) and, if necessary, adjust statistically Evaluate and capture the fidelity of all study components to support interpretation and rigorous study reporting
Outcomes	Ideal:	All aspects of ‘CORE Research’ should be designed to evaluate the impact of the intervention on a primary or co-primary clinically relevant outcome. Hard end-points, including mortality, major cardiovascular events and hospitalizations, should be considered when feasible. Adopt gold standard methods to evaluate key secondary and tertiary outcomes.
	Minimum:	The primary outcome needs to be specifically tailored to account for its unique characteristics. Make every effort to identify, characterize, and evaluate the influence of external factors when analysing, interpreting, and discussing study results.
CORE intervention design		
Design and evaluation	Ideal:	Elements of all CORE intervention components should be designed and defined according to the expanded FITT-P principles* and appropriately monitored to collect the information required to facilitate interpretation, reporting and reproducibility *The theories underlying the FITT-P principles are technically intervention agnostic—meaning the can be adapted to describe characteristics of many types of medical and behavioural therapies.
	Minimum:	All programmes should follow the local standards for cardiac rehabilitation and include tailored exercise prescription, nutritional and psychosocial support, behavioural change, and patient education. Programmes should include standard evaluations measures for attendance, adherence and potential outcomes.
Personalization	Ideal:	Tailoring to relevant factors, including medical history, comorbidities, baseline testing results and study objectives Use FITT-P when feasible FOCUS POINT: Intervention Intensity Prescribed using gold-standard physiological parameters (e.g. VO_2_peak or peak power output (PPO)). A combination of workload-defined training targets (e.g. PPO) with percentages of heart rate reserve and/or RPEs may be the most rigorous method for monitoring exercise intensity, especially in patients receiving anticancer therapy
	Minimum:	Tailor intervention components using the most accurate and reliable method available and discuss methods used in the context of their advantages and limitations. Prioritize understanding and implementing strategies to ensure the intervention is feasible (e.g. financial barriers, convenient times, etc.) and enjoyable (e.g. well-tolerated and in a supportive environment)
CORE study reporting		
General reporting	Minimum:	Tailor intervention components using the most accurate and reliable method available and discuss methods used in the context of their advantages and limitations Prioritize understanding and implementing strategies to ensure the intervention is feasible (e.g. financial barriers, convenient times, etc.) and enjoyable (e.g. well-tolerated and in a supportive environment)
Trial reporting	Minimum:	Use reporting guidelines for medical RCTs [e.g. the Consolidated Standards of Reporting of Clinical Trials (CONSORT) 2010 statement,^149^] non-pharmacological RCTs (e.g. CONSORT NPT,^150^) harms reporting (e.g. CONSORT Harms,^151^) and intervention reporting (e.g. TIDier^152^)
Intervention reporting	Minimum:	Use and adapt validated and standardized tools for reporting all intervention components *For example*: The CERT is a 16-item checklist characterizing exercise interventions across seven sections: what (materials); who (provider): how (delivery): where (location); when, how much (disease) tailoring (what, how) and how well (compliance/planned and actual)^153^ Exercise Intensity Monitoring and Reporting: *Investigators* should track and report the average achieved intensity (using any available method) and delivery duration for all core-related interventions and co-interventions, even if the data is not combined to calculate and report adherence The achieved workloads during treadmill and bike-based sessions should be estimated using ASCM prediction equations and/or directly measured from the training equipment The intensity of the data is then multiplied by the duration of time spent training or training at each workload, summed (for HIIT sessions), and covered into metabolic equivalents to calculate relative dose intensity
Safety reporting	Ideal:	Exercise Harms Report Method (ExHaRM) inventory defines exercise-related harms as all undesirable physical, psychological, economic, or social consequences (covering incidences, experiences, occurrences) related to a given an individual's participation in exercise. ExHaRM or a comparable tool should be consistently used to guide harms reporting for every intervention component tested within CORE and CORE-relevant studies
	Minimum:	Harms data should be systematically documented throughout the study period in both/all arms (if they exist), including the frequency, severity, and likely causes. As recommended by the CONSORT Harms extension, these findings should be summarized and reported in primary publications and clinical trial registries to provide a comprehensive picture of risks and benefits for all interventions.

### CORE data sharing recommendations

Broad clinical trial data sharing strategies (e.g. via clinical trial registries) have been developed and are gradually being adopted to increase transparency and accelerate discovery across research sectors. Expanding this approach to support progress in CORE and CORE-relevant research areas can drive discovery by helping investigators overcome the challenges associated with studying small homogeneous populations to detect smaller, yet clinically relevant, intervention effects. To this end, the ICOS CORE WG is developing an online research platform to promote international collaboration and data sharing (https://ic-os.org/committees/exercise/). *Ideally*, investigators will use the platform to (i) list their labs, contact information, and ongoing/completed trials, (ii) promote and seek out opportunities for international collaboration, and (iii) provide links to their study records (e.g. clinical trial registry pages), data repositories, and relevant manuscripts. *At minimum*, investigators should post-clinical trial notifications and publications and, where appropriate, share summary-level data, code, or regression outputs on reasonable request. The CORE platform will include a running list of clinical trials updated via clinical trial registries.

Notably, one of the primary benefits of data sharing within a research community is the ability to consolidate and synthesize data from multiple sources. However, such efforts are currently hindered by the large heterogeneity in the various factors described above. One challenge faced by CORE researchers, and behavioural scientists in general, is the ability to appropriately capture the complexities of diverse assessment methods, intervention designs, and intervention delivery/fidelity metrics to facilitate the interpretation, reproducibility, and synthesis of evidence across studies. Thus, the standardization of research methods and reporting practices described above is critically important to efficiently use the limited financial support available to the community, accelerate discovery, evaluate the cost-effectiveness of the CORE model across diverse healthcare settings and geographic regions, and, ultimately, facilitate the widespread implementation of CORE services.

## CORE’s potential benefits across stakeholders

The widespread implementation of CORE has the potential to have a broad impact for multiple stakeholders, here we touch on the potential benefits for the individual, healthcare providers, and systems.

### Benefits for survivors

Cardiac rehabilitation is a widely available multimodal care model with well-established health benefits in primary, predominantly secondary, and tertiary CVD prevention contexts. Evidence from meta-analysis and Cochrane reviews suggests that cardiac rehabilitation improves cardiorespiratory fitness,^152^HRQoL,^154^ psychosocial distress (e.g. depression, anxiety),^155^ metabolic outcomes,^156^ reduces the risk of myocardial infarction,^149^ and is associated with reductions in all-cause (−30%)^150^ and CVD-related (−41%)^150^ hospitalizations, and all-cause (range: −13% to −47%)^149,151,157–159^ and CVD-related mortality (range: −26% to −58%) across individuals living with various cardiac diseases.^149,151,157–159^ Studies evaluating cardiac rehabilitation's primary intervention components as standalone interventions (e.g. physical activity counselling, behaviour change, moderate-to-vigorous aerobic exercise training) have demonstrated they are safe and also significantly improve or mitigate declines in diverse physiological and psychosocial outcomes across relevant clinical populations (e.g. cancer, heart failure, kidney disease, depression, and CVD risk populations).^160–163^ However, some outcomes have been shown to respond better to specific stimuli in select populations. For example, there is evidence that higher-intensity exercise training during cardiac rehabilitation is as safe as moderate-intensity exercise in patients with coronary artery disease,^164,165^ leads to similar, if not greater benefits for selected physiological outcomes in some^164,165^ but not all^160^ populations, and may improve long-term retention of protective levels of physical activity behaviour.^166^ The latter finding is important as sustaining health behaviours following the completion of supervised cardiac rehabilitation is associated with improved HRQoL^167^ and survival.^168^ Unfortunately, the time, effort, and expertise required to deliver effective exercise- and nutrition-based interventions, education, and behavioural support far exceed the practical abilities and training of most front-line HCPs. If the observed benefits of cardiac rehabilitation do translate to CORE, the most impactful action HCPs can take towards supporting the long-term health of cancer patients and survivors, beyond cancer elimination, is referring them to CORE. However, cardio-oncology survivors’ demographic and health profiles may differ in important ways from that of traditional cardiac patients (e.g. younger age, toxic treatment exposures, employment status). Therefore, whether similar findings are replicable in cardio-oncology survivors requires confirmation.

### Benefits for healthcare providers

The integration of CORE within the current cardio-oncology care model and/or existing cardiac rehabilitation programmes may help address many of the described theoretical and practical care challenges for HCPs. For instance, CORE programmes could help augment screening for CVD risk and functional impairments via routine clinical assessments, including functional testing or CPET. The suggested CORE model is well suited to providing patients with dedicated and high-quality education and behavioural support for CVD risk factor management outside of cardio-oncology clinics and for supporting the broader cancer treatment and symptom management care priorities of patients. Participation in CORE would likely also support the long-term CVD-related care objectives of HCPs (e.g. improving CVD risk factor management and reducing CVD events) via the synergistic effects of concurrent exercise, nutrition, and pharmacologic-based therapies. Inferring from the cardiac rehabilitation literature, the specific combination of treatment with these potent multimodal therapies and exposure to high-quality education and behavioural support may confer considerable near- and long-term health benefits to cancer survivors by causing favourable near-term adaptations across the cardiovascular system and providing survivors with the knowledge and self-governed expertise to maintain protective health behaviours in the years and decades following treatment.

### Benefits for the healthcare system and healthcare utilization

There is a large body of evidence demonstrating the cost-effectiveness of exercise-based cardiac rehabilitation that bears consideration in the context of CORE. A recent systematic review^169^ comparing exercise-based cardiac rehabilitation to no cardiac rehabilitation reported favourable cost-effectiveness ratios for cardiac rehabilitation ranging from $1065 to $71 755 per quality-adjusted life year across the seven included studies.^170–176^ The identified drivers of cost-effectiveness include subsequent event and hospitalization risk, costs of interventions and hospitalizations, and required utilities.^169^ Indeed, as highlighted above, the recently published cost-effective analysis of the CORE trial provides the first evidence of the cost-effectiveness of a multidisciplinary 8-week intervention targeted at cancer survivors with increased cardiovascular risk. The programme was found to be cost-effective when implemented in high-risk cardiovascular cancer survivors when compared with a usual care model of community-based exercise, considering the calculated incremental cost-effectiveness ratio cost of €1383.24 per additional quality-adjusted life year.^71^ These findings from the CORE study provide cause for optimism that similarly to cardiac rehabilitation this model of care will be found to be cost-effective and can improve important patient outcomes with individual and societal implications (e.g. reduced years of potential life lost, delays in return to work, and long-term disability support).

In summary, early evidence from CORE research combined with the considerable indirect evidence from the cardiac rehabilitation literature suggests that adopting and integrating CORE into the cardio-oncology care model has tremendous potential to positively impact numerous patient, HCP, and healthcare system-level outcomes. Despite early promise, limited evidence confirms that exercise-based therapies have broad benefits for cancer survivors with or at risk of CTRCD.

### Summary

CORE incorporates a model like cardiac rehabilitation programmes, including medical evaluation, prescriptive exercise, cardiovascular risk factor modification, education, counselling, and nutritional support. This intervention potentially has diverse and complementary patient-, HCP-, and system-level benefits and may be a leading strategy to address the current care gaps and optimize long-term cardiovascular outcomes for oncological patients and survivors. We have defined key concepts, evidence gaps, and standards to support the design and conduct of critical CORE research, efficient dissemination and use of CORE research, and overcome many current research and care challenges. Close collaboration of CORE stakeholders (e.g. oncologists, cardiologists, primary care physicians, nursing, exercise physiologists/therapists, nutritionists, etc.) is required to accelerate discovery, improve the quality (e.g. rigour and reporting) and scope (e.g. targeting high risk and highly vulnerable populations) of CORE research, and support widespread implementation in the field.

## Supplementary Material

ehaf100_Supplementary_Data

## Declarations

### Data Availability

No data were generated or analysed for this manuscript.
