# Supplementary material for: Cardio-oncology rehabilitation and exercise: evidence, priorities, and research standards from the ICOS-CORE working group
Source: Eur Heart J. 2025 Feb 28;46(29):2847–65. doi: 10.1093/eurheartj/ehaf100 (PMC12314747; doi:10.1093/eurheartj/ehaf100)
Supplement: ehaf100_Supplementary_Data [file ehaf100_supplementary_data.docx]

**Title: Cardio-Oncology Rehabilitation and Exercise: Evidence, Priorities, and Research Standards from the ICOS-CORE Working Group**

**Authors:** Scott C. Adams^1^, Fernando Rivera-Theurel^2-4^, Jessica M. Scott^5^, Michelle B. Nadler^6,7^, Stephen Foulkes^8^, Darryl Leong^9^, Tormod Nilsen^10^, Charles Porter^11^, Mark Haykowsky^8^, Husam Abdel-Qadir^2,3,12^, Sarah C. Hull^13, 14^, Neil M. Iyengar^15,16^, Christina M. Dieli-Conwright^,17,18,19^, Susan F. Dent^20^, Erin J. Howden^21^

**SUPPLEMENTAL MATERIAL**

**CORE Study Design Standards**

**Patients / Population:** ***Ideally***, studies should selectively recruit well-defined groups of participants with cardiovascular sequelae or risk thereof. When recruited groups are more heterogeneous, it is strongly recommended that stratification factors be evaluated, discussed and considered by the entire multi-disciplinary team to ensure a balance of cardiac risk. The ICOS CORE research registry is a resource to seek out collaborators and opportunities to promote and support new and ongoing multicenter studies. ***At minimum***, in settings or circumstances where these strategies are not feasible, investigators should consider conducting rigorous pilot/feasibility studies. These studies can optimize their utility and impact to accelerate discovery in the field by carefully characterizing, evaluating and reporting the most critical aspects of their design and implementation (e.g., participant recruitment, enrollment and retention rates, intervention fidelity) to inform the development and conduct of highly feasible and adequately powered clinical trials. Investigators across settings should also provide complete descriptions of essential participant characteristics required for interpreting and reproducing the findings of studies, including details of (1) specific cancer diagnoses, dates and staging, (2) specific doses and scheduling of various anti-cancer therapies, and (3) relevant medical characteristics (e.g., pre-cancer and familial CVD histories, presence of traditional and novel CVD-related risk factors, biomarkers and comorbidities at baseline and follow-up).

**Interventions: *Ideally***, all components of multimodal CORE interventions should be specifically designed to directly target or indirectly support improvements in cardiovascular and metabolic function, physical and psychosocial health, quality of life or survival-related outcomes. The exercise component(s) of interventions should be personalized and progressed for each participant via data derived from their baseline or interim CPET (e.g., % of achieved VO_2peak_, metabolic equivalents, or peak power output) and/or 1-10 RM testing (e.g., % of maximum strength per exercise) and could be delivered either as face-to-face, remotely or hybrid program. Other CORE intervention components should be personalized according to best practices within each field. For example, pharmacotherapies should be used according to current clinical guidelines and where appropriate patients should receive education and support to optimize medication adherence. Similarly, nutrition-based intervention components should be personalized and delivered by registered dietitians. Behavioural support and education intervention components should be tailored to the needs of each individual patient, with a multi-component approach considered best-practice to optimise patient outcomes. Indeed, tailored behaviour change and behavioural support can positively impact all elements of the multi-component CORE intervention approach and should be a key component of interventions. However, we acknowledge that not all investigators have access to the type of specialized equipment / technology and personnel required to adopt gold-standard methods for intervention personalization. Therefore, ***at minimum***, investigators should personalize CORE-relevant intervention elements as best they can based on their local cardiac rehabilitation resources, reporting the detailed methods used and their relative strengths and limitations. For example, exercise investigators can tailor intervention intensity as best they can within their means (e.g., % of achieved or estimated maximal heart rate or via ratings of perceived exertion) and carefully document and report all relevant intervention-related fidelity metrics, including attendance, compliance, adherence, interruptions, adjustments, and discontinuations (1). This applies to both centre-based and remotely delivered programs. The primary pillars of cardiac rehabilitation interventions (e.g. exercise, nutrition, education) are well established. However, regional and institutional-level differences in facilities, equipment, personnel, and funding preclude the possibility of establishing universal minimum standards for patient assessment, treatment, and data collection. CORE programs and research activities face similar practical challenges that undermine their rigour and reproducibility. Therefore, investigators should provide the details required to replicate and implement all components of the tested CORE interventions and, where possible, provide evidence of fidelity (e.g. number of education sessions complete; compliance with nutrition intervention). Furthermore, all investigators should provide clear theoretical rationale and practical intervention descriptions of how each intervention component is appropriately dosed and tailored to influence the outcomes of interest. See Study Reporting for related recommendations.

**Comparisons:** ***Ideally***, intervention studies should be randomized and adhere to clinical trial best practices – involving the inclusion of appropriate and well-characterized comparison groups, as appropriate for efficacy (e.g., active or passive control group(s)) or superiority (e.g., standard of care or self-directed intervention arms) trial designs. However, we appreciate that not all studies occur in medical, and population centers conducive to implementing these more rigorous, multi-arm study designs. Thus, ***at minimum***, investigators should make every effort to anticipate, control for (by study design), and report key intervention confounders (e.g., non-trial physical activity) and, where necessary, adjust statistically. Furthermore, as appropriate, the complete methods for (1) participant randomization, allocation, and blinding of outcome assessors (where possible) and (2) investigator and analysis blinding (even in non-randomized and single-arm trials), as well as fidelity metrics for how well these procedures were implemented should be reported for all studies. See Study Reporting for related recommendations.

**Outcomes:** The primary outcome represents a critical convergence point within study designs because the population-, comparison-, intervention-, and statistics-related aspects of trials should all be specifically tailored to account for its unique characteristics. **‘CORE Research’ – *Ideally***, all aspects of ‘CORE studies’ should be designed to evaluate the impact of a CORE-based intervention on a primary or co-primary clinically relevant cardiovascular outcomes. It is also important to note that different cancer patients and anti-cancer treatments will experience/result in different cardiotoxicities, which is an important consideration when determining relevant outcomes. Ultimately, hard end-points, including mortality, major adverse cardiovascular events, and hospitalizations, require evaluation, though historically, there have been few exercise and lifestyle interventions which have evaluated these outcomes (2). However, the causes of cancer-related cardiovascular sequelae experienced by patients are often multifactorial. Thus, it is also important to carefully select and evaluate key secondary and tertiary outcomes (e.g., established or theoretical confounding variables [e.g. non-trial physical activity, nutrition, or medication changes]) via their respective gold-standard assessment techniques to facilitate the interpretation of the main findings and situate the main findings within their broader biopsychosocial and healthcare system/societal context. **‘CORE-Relevant Research’ –** It is also important to acknowledge the potential contributions of investigators who may not be primarily interested in testing a multimodal CORE-based intervention model or focusing on a cardiovascular endpoint as their primary outcome given their specific research expertise (e.g., nutrition- or behaviour-focused scientists), or be capable of doing so due to financial or practical restraints. Nonetheless, these studies can still provide critically important insight into key elements of CORE-based interventions. In these settings, ideally, investigators will provide clear details regarding the CORE-related rationale and theoretical underpinnings of their work and similarly adopt best practices in study design and gold standard assessment methods, control for the effects of known and suspected confounders, and discuss their findings within the context of cardio-oncology patient care. However, there are often financial, physical, or human resource restraints that limit the capacity of investigators to comprehensively characterize all the established and likely confounders for given outcomes. Therefore, ***at minimum***, investigators should make every effort to identify and account for the influence of these factors or disclose their inability to account for these factors when analyzing, interpreting, and discussing their findings. For example, study teams that do not have access to wrist-worn wearable devices can still account for non-trial physical activity via validated questionnaires or asking participants to keep a physical activity logbook. See Study Reporting for related recommendations.

**CORE Intervention Design Standards**

The primary elements of CORE interventions (e.g., exercise, nutrition, education) and associated support activities (e.g., behavioural support) should be (1) personalized, (2) defined according to the expanded FITT-P principles (i.e., Frequency, Intensity, Time, Type, and Progression), and (3) reported with enough detail to be interpretable and reproducible. The FITT-P principles were initially conceived to facilitate and standardize exercise prescriptions; however, the theories underlying the FITT-P principles are technically intervention agnostic – meaning they can be adapted to describe the characteristics of many types of medical and behavioural therapies. Therefore, investigators should plan, deliver, and report the fidelity of all primary CORE intervention components using the FITT-P principles.

Personalization is arguably the most important consideration when optimizing the safety, tolerability, and efficacy of exercise and lifestyle interventions. Personalization involves tailoring interventions for all relevant factors, including medical history, current comorbidities, baseline testing results, and study objectives. Theoretically, all CORE intervention components can be tailored using the FITT-P principles. In exercise, intensity is often the most important FITT-P principle to personalize. However, exercise intensity is one of the most challenging FITT principles to standardize and monitor in practice. ***Ideally***, exercise intensity should be prescribed and monitored based on comparatively accurate and reliable physiological or performance parameters (e.g., VO_2_peak or peak power output (PPO)). Prescriptions based on percentages of directly measured VO_2_peak or PPO are preferred because they are less susceptible to the influence of transient environmental factors or conditions (e.g., hydration or sleep status) compared to prescriptions using percentages of peak heart rate or RPE. However, using VO_2_peak to monitor exercise intensity during training sessions is impractical in most settings as it requires access to specialized equipment and personnel, and can be burdensome for the patient. Percentages of estimated or achieved peak heart rates are commonly used to prescribe and monitor exercise intensity. However, exercise prescriptions based on age-predicted heart rate values are less rigorous as estimated peak heart rates may vary by up to 10-12 beats-per-minute (3). Further, using heart rate to define and monitor target training intensities may not be reliable in patients receiving anticancer therapy or in those with suspected cardiac autonomic dysfunction (4) or receiving treatment with rate limiting medications. In general, using a combination of workload defined training targets (e.g., PPO) with percentages of heart rate reserve and/or RPEs may be the most rigorous method for prescribing and monitoring exercise intensity, especially in patients receiving anticancer therapy. The importance of tailoring exercise intensity is becoming more widely recognized given the emerging evidence that outcomes may be differentially impacted by specific exercise intensities (e.g., high-intensity aerobic exercise elicits greater improvements in VO_2_peak while moderate-intensity exercise causes greater improvements in glucose metabolism) (5). If validated, these findings would help clinical and academic interventionists personalize their exercise programs to their patients' specific deficits and comorbidities. ***At minimum***, investigators should make every effort to tailor intervention components using the most accurate and reliable method available and discuss the methods used in the context of their advantages and limitations. Investigators should also prioritize understanding and implementing strategies to ensure the interventions are as feasible (e.g., offered at convenient times; minimal financial barriers to engagement) and enjoyable (e.g., well-tolerated prescriptions and supportive environments) as possible to support optimal intervention adherence and long-term maintenance of the important and protective behaviours.

Other important elements to consider when testing CORE and CORE-relevant interventions are somewhat context- or setting-dependent. For example, the duration of cardiac rehabilitation programs differs between countries. In Canada, cardiac rehabilitation programs typically offer 6 to 12 months of service, whereas, the typical program durations in the US and Australia vary between 6 and 36 sessions. Similarly, there is a great deal of heterogeneity in how programs are delivered (e.g., supervised on-site, remotely supervised, or self-directed) within and between different regions and countries, particularly in the post-COVID-19 era. Until optimal intervention-specific doses and delivery methods are established for specific populations and outcomes, investigators may be limited to testing interventions that are practically designed to reflect the structure of their local cardiac rehabilitation program.

**CORE Study Reporting Standards**

Incomplete study reporting is a major impediment to progress in this field. Recent reviews assessing the quality of CORE-based studies (6) and comparing the quality of exercise and pharmacotherapy trials across clinical populations (7) reported major deficiencies in the completeness of reporting critical elements of study design and conduct (e.g., fidelity), intervention design and delivery (e.g., adherence), and patient safety, as well as a moderate-to-high degree of risk-of-bias across studies. Unfortunately, similar issues exist within the cardiac rehabilitation evidence base. For example, a review of cardiac rehabilitation trials (8) reported that only 15% of studies provided adequate intervention descriptions, according to the Template for Intervention Description and Replication (TIDieR) checklist (9). These and related reviews all caution that the pervasive incomplete reporting of critical study and intervention details is a primary factor limiting the translation and implementation of therapies that could dramatically improve outcomes for patients (6, 7, 9-11).

**Reporting Guidelines:** Notably, these reporting quality- and risk-of-bias-related limitations are not unique to behavioural interventions, with similar limitations also being reported in trials of chemotherapy (12), surgeries, and medical devices. To address these issues, organizations have developed reporting guidelines for medical RCTs (e.g., the Consolidated Standards of Reporting of Clinical Trials (CONSORT) 2010 statement (13)), non-pharmacological RCTs (e.g., CONSORT-NPT (14)), harms reporting (e.g., CONSORT-harms (15)), and intervention reporting (e.g., TIDieR (9)). Together, these guidelines provide important direction to support investigators in planning and reporting critical trial elements. However, the nuanced details required to interpret, replicate, and translate the findings of complex intervention models, like CORE, are not adequately covered by these frameworks.

**Intervention Reporting:** To address some of the issues related to the reporting of exercise interventions, an international Delphi study was undertaken to develop a standardized method for reporting. The resultant Consensus on Exercise Reporting Template (CERT) is a 16-item checklist that prompts for exercise descriptions across seven sections: what (materials); who (provider): how (delivery): where (location); when, how much (disease) tailoring (what, how) and how well (compliance/planned and actual) (16). The CERT builds on earlier recommendations from TIDieR to include more information about the type of exercise, dosage, intensity, frequency and supervision requirements. In addition, when an individualized rather than a ‘one-size fits all’ programme is tested, CERT requires that investigators report specific details of how exercises are tailored to the needs and abilities of individual participants. This working group's consensus is that CERT, like the FITT-P principles, should be adopted and adapted by CORE investigators to characterize all aspects of CORE multi-modal treatment model. In their paradigm-shifting paper, Nilsen and colleagues (1) adapted methods used to monitor performance within athletes and drug tolerability within oncology trials to enhance the reporting of exercise intervention fidelity by proposing a comprehensive set of improved and/or novel methods for evaluating and reporting of key outcomes, including intervention adherence (i.e., relative dose intensity – the ratio of achieved exercise dose to total planned exercise dose) and an array of intervention tolerability metrics (i.e., the number and percent of attendance; completed follow-up assessments; permanent treatment discontinuations; treatment interruptions; dose modifications; early session terminations; and, pre-treatment intensity modifications). Aerobic exercise adherence is a composite outcome wherein the dose is calculated by summing the time spent working at each achieved exercise intensity during a session and dividing that sum by the planned dose of exercise for each session, quantified as metabolic equivalents. A similar approach is recommended to assess and report resistance exercise adherence (i.e., exercise relative dose intensity, dose modification and quantified as total training volume in kilograms (17)). Unfortunately, intervention dose and adherence are systematically mis- or under-reported aspects of exercise, diet, and behavioural interventions in oncology, precluding the characterization of dose-response relationships. Thus, investigators are encouraged to use the best available methods (e.g., Nilsen et al. (1)) to quantify and report participant adherence to all CORE intervention elements.

Investigators across CORE-relevant research disciplines (e.g., exercise, nutrition) should be aware, and carefully consider the limitations, of their chosen method of quantifying intervention dose and adherence. In an aerobic exercise context, ***ideally*** the achieved workloads during treadmill and bike-based exercise sessions would be estimated using ACSM prediction equations and/or directly measured from the training equipment. The intensity data is then multiplied by the duration of time spent training (for MICE sessions) or training at each workload, summed (for HIIT sessions), and converted into metabolic equivalents to calculate relative dose intensity. Similarly, discipline-specific methods can be adopted when quantifying the achieved doses of pharmacotherapy, resistance exercise, nutrition, education, and behavioural interventions to provide critical insight into the fidelity of how completely these interventions were delivered and to facilitate the conduct of dose-response analyses. However, dose-quantification strategies for all intervention types will be variably accurate and informative across settings and uses. Therefore, ***at minimum***, investigators should track and report the average achieved intensity (using any available method) and delivery duration for all CORE-related interventions and co-interventions, even if they do not combine the data to calculate and report adherence.

**Safety Reporting:** Adverse event reporting is a critical part of clinical research. The CONSORT Harms extension provides specific guidance for defining and reporting of adverse events in clinical trials (18). Unfortunately, harms data is systemically underreported in CORE and CORE-relevant trials. Therefore, the true incidence of harms that may be attributable to any of CORE’s constituent intervention components are largely unknown. Indeed, when performing interventional studies, most studies are designed to test the *efficacy* of an intervention and are therefore underpowered to detect differences in adverse events. This should not, however, prevent investigators from rigorously documenting and reporting adverse events. Recently, Spence and colleagues (10) proposed a framework for the exercise oncology field that could be adopted for CORE research. The Exercise Harms Report Method (ExHaRM) defines exercise-related harms as all undesirable physical, psychological, economic, or social consequences (covering incidences, experiences, occurrences) related to a given an individual’s participation in exercise. This four-step process involves (1) monitoring of adverse events by systematic and non-systematic means; (2) assessing and recording the key details of each adverse event, including type, frequency, severity, causality, impact on the individual and study; (3) a review of all harms by a study oversight panel to discuss and document attribution for all adverse events; and, (4) conducting analysis and reporting of harms frequencies, rates, and relevant details for all adverse events regardless of attribution. ***Ideally****,* this ExHaRM-defined process should be implemented for all CORE and CORE-relevant studies. ***At minimum***, harms data should be systematically documented throughout the study period in both/all arms (if they exist) including the frequency, severity, and likely causes. These findings should be summarised and reported within primary publications and through clinical trial registries to provide a comprehensive picture of risks and benefits for all intervention, as recommended by the CONSORT-Harms extension.

**References**

1. Nilsen TS, Scott JM, Michalski M, Capaci C, Thomas S, Herndon JE, 2nd, et al. Novel Methods for Reporting of Exercise Dose and Adherence: An Exploratory Analysis. Med Sci Sports Exerc. 2018;50(6):1134-41.

2. O'Connor CM, Whellan DJ, Lee KL, Keteyian SJ, Cooper LS, Ellis SJ, et al. Efficacy and safety of exercise training in patients with chronic heart failure: HF-ACTION randomized controlled trial. JAMA. 2009;301(14):1439-50.

3. Fairbarn MS, Blackie SP, McElvaney NG, Wiggs BR, Pare PD, Pardy RL. Prediction of heart rate and oxygen uptake during incremental and maximal exercise in healthy adults. Chest. 1994;105(5):1365-9.

4. Moslehi JJ. Cardiovascular toxic effects of targeted cancer therapies. N Engl J Med. 2016;375(15):1457-67.

5. Weston KS, Wisloff U, Coombes JS. High-intensity interval training in patients with lifestyle-induced cardiometabolic disease: A systematic review and meta-analysis. Br J Sports Med. 2014;48(16):1227-34.

6. Fakhraei R, Peck BSS, Abdel-Qadir H, Thavendiranathan P, Sabiston CM, Rivera-Theurel F, et al. Research Quality and Impact of Cardiac Rehabilitation in Cancer Survivors: A Systematic Review and Meta-Analysis. JACC CardioOncol. 2022;4(2):195-206.

7. Adams SC, McMillan J, Salline K, Lavery J, Moskowitz CS, Matsoukas K, et al. Comparing the reporting and conduct quality of exercise and pharmacological randomised controlled trials: a systematic review. BMJ Open. 2021;11(8):e048218.

8. Abell B, Glasziou P, Hoffmann T. Reporting and replicating trials of exercise-based cardiac rehabilitation: do we know what the researchers actually did? Circ Cardiovasc Qual Outcomes. 2015;8(2):187-94.

9. Hoffmann TC, Glasziou PP, Boutron I, Milne R, Perera R, Moher D, et al. Better reporting of interventions: template for intervention description and replication (TIDieR) checklist and guide. BMJ. 2014;348:g1687.

10. Spence RR, Sandler CX, Jones TL, McDonald N, Dunn RM, Hayes SC. Practical suggestions for harms reporting in exercise oncology: the Exercise Harms Reporting Method (ExHaRM). BMJ Open. 2022;12(12):e067998.

11. Nieuwlaat R, Schwalm JD, Khatib R, Yusuf S. Why are we failing to implement effective therapies in cardiovascular disease? Eur Heart J. 2013;34(17):1262-9.

12. Duff JM, Leather H, Walden EO, LaPlant KD, George TJ. Adequacy of Published Oncology Randomized Controlled Trials to Provide Therapeutic Details Needed for Clinical Application. JNCI: Journal of the National Cancer Institute. 2010;102(10):702-5.

13. Moher D, Hopewell S, Schulz KF, Montori V, Gotzsche PC, Devereaux PJ, et al. CONSORT 2010 explanation and elaboration: updated guidelines for reporting parallel group randomised trials. BMJ. 2010;340:c869.

14. Boutron I, Altman DG, Moher D, Schulz KF, Ravaud P, Consort NPT Group. CONSORT statement for randomized trials of nonpharmacologic treatments: A 2017 update and a CONSORT extension for nonpharmacologic trial abstracts. Ann Intern Med. 2017;167(1):40-7.

15. Ioannidis JP, Evans SJ, Gotzsche PC, O'Neill RT, Altman DG, Schulz K, et al. Better reporting of harms in randomized trials: an extension of the CONSORT statement. Ann Intern Med. 2004;141(10):781-8.

16. Slade SC, Dionne CE, Underwood M, Buchbinder R, Beck B, Bennell K, et al. Consensus on Exercise Reporting Template (CERT): Modified Delphi Study. Phys Ther. 2016;96(10):1514-24.

17. Fairman C, Nilsen TS, Newton RU, Taaffe DR, Spry N, Joseph D, et al. Reporting of resistance training dose, adherence, and tolerance in exercise oncology. 2020.

18. Better Reporting of Harms in Randomized Trials: An Extension of the CONSORT Statement. Annals of Internal Medicine. 2004;141(10):781-8.
